# Colonic Mucosal Microbiota in Colorectal Cancer: A Single-Center Metagenomic Study in Saudi Arabia

**DOI:** 10.1155/2018/5284754

**Published:** 2018-05-16

**Authors:** Ahmed O. Alomair, Ibrahim Masoodi, Essam J. Alyamani, Abed A. Allehibi, Adel N. Qutub, Khalid N. Alsayari, Musaad A. Altammami, Ali S. Alshanqeeti

**Affiliations:** ^1^Gastroenterology & Hepatology Department, King Fahad Medical City, Riyadh, Saudi Arabia; ^2^Department of Medicine, College of Medicine, University of Taif, Taif, Saudi Arabia; ^3^National Center for Biotechnology, King Abdulaziz City for Science and Technology (KACST), Riyadh, Saudi Arabia; ^4^National Blood & Cancer Center, Riyadh, Saudi Arabia

## Abstract

**Background and Aim:**

Because genetic and geographic variations in intestinal microbiota are known to exist, the focus of this study was to establish an estimation of microbiota in colorectal cancer (CRC) patients in Saudi Arabia by means of metagenomic studies.

**Methods:**

From July 2010 to November 2012, colorectal cancer patients attending our hospital were enrolled for the metagenomic studies. All underwent clinical, endoscopic, and histological assessment. Mucosal microbiota samples were collected from each patient by jet-flushing colonic mucosa with distilled water at unified segments of the colon, followed by aspiration, during colonoscopy. Total purified dsDNA was extracted and quantified prior to metagenomic sequencing using an Illumina platform. Satisfactory DNA samples (*n* = 29) were subjected to metagenomics studies, followed by comprehensive comparative phylogenetic analysis. An equal number of healthy age-matched controls were also examined for colonic mucosal microbiota.

**Results:**

Metagenomics data on 29 patients (14 females) in the age range 38–77 years were analyzed. The majority 11 (37%) of our patients were overweight (BMI = 25–30). Rectal bleeding was the presenting symptom in 18/29 (62%), while symptomatic anemia was the presenting symptom in 11/29 (37%). The location of colon cancer was rectal in 14 (48%), while cecal growth was observed in 8 (27%). Hepatic flexure growth was found in 1 (3%), descending colonic growth was found in 2 (6%), and 4 (13%) patients had transverse colon growth. The metagenomics analysis was carried out, and a total of 3.58G reads were sequenced, and about 321.91G data were used in the analysis. This study identified 11 genera specific to colorectal cancer patients when compared to genera in the control group. *Bacteroides fragilis* and *Fusobacterium* were found to be significantly prevalent in the carcinoma group when compared to the control group.

**Conclusion:**

The current study has given an insight into the microbiota of colorectal cancer patients in Saudi Arabia and has identified various genera significantly present in these patients when compared to those of the control group.

## 1. Introduction

Metagenomics is a molecular method of culture-independent microbiology, in which genetic material recovered directly from environmental samples is studied. It has emerged as one of the most robust sequence-driven approaches for studying the composition and the genetic potential of the mucosal gut microbiota. Metagenomics analysis has begun to demonstrate the breadth of the functional and metabolic potential of microbes. It has been used to demonstrate significant metabolic discrepancies between diseased and healthy individuals. Although the intestinal microbiota in individuals reflects great variations among people according to their age, geographic origin, state of health, and variations in diet, it tends to remain stable over long periods [1].

An alteration in gut microbiota can cause the development of inflammation within the colon, and such inflammation is implicated in colonic neoplastic development. Although the precise mechanisms through which the microbiota is involved in cancer development remain elusive, the message is, however, clear: the microbiota contributes to cancer risk by influencing some fundamental host processes [2]. This implies that modifiable risk factor interventions to modulate gut microbiota can contribute to decreasing the morbidity and mortality rates in colon cancer.

The risk of developing colorectal cancer (CRC) varies markedly between and within populations and geographical regions [3]. Accordingly, various aspects of carcinogenesis in colon cancer have been addressed in Saudi Arabia by different investigators from time to time [4–8], but to the best of our knowledge and after a literature search, there appears to be no existing data regarding the role of microbiota in CRC in this part of the globe. Hence, we were prompted to undertake this study, first of its kind in Saudi Arabia, in which we have tried to introspect the role of mucosal intestinal microbiota in CRC patients.

It will be prudent to mention that the mucosal microbiota lives closer to the intestinal epithelium when compared to the luminal microbiota, and conceivably, it would be interacting more directly with the host immune system than would the luminal/fecal bacteria. It is quite possible that mucosal microbiota might be more directly involved in inducing colon carcinogenesis. In addition, the availability of nutrients in the mucus layer of the epithelium is also entirely different from that in the gut lumen environment. Substantial differences in mucosal and fecal microbial composition have been shown to exist [9, 10]. Hence, we choose mucosal samples and not fecal samples for this metagenomic study in CRC patients. The microbiota was compared with that in age- and gender-matched controls.

## 2. Material and Methods

This study was conducted in full compliance with the guidelines for good clinical practice of the World Medical Assembly Declaration of Helsinki and the research guidelines of the King Fahad Medical City (KFMC), Riyadh. The study was approved both by the Ethics Committee of King Abdulaziz City for Science and Technology and KFMC, Riyadh.

### 2.1. Inclusion Criteria for Study Participants

During the study period (July 2010 and November 2012), all colonoscopy patients at King Fahad Medical City (KFMC), Riyadh, were asked to provide specific written consent for possible inclusion in this study. Samples collected during the colonoscopy procedures in the following types of patient were then processed.

(1) Cases

(a) Adult patients with diagnosed colorectal cancer whose diagnoses were based on endoscopic and histological criteria.

(2) Controls

(a) Patients undergoing screening colonoscopic examination with normal colonoscopic procedures.
(b) Patients undergoing colonoscopic procedures for lower GI bleed who had been proved to have hemorroids and anal fissures.
(c) Patients with abdominal pain undergoing the colonoscopic procedure and who proved to have a normal colonoscopic examination

### 2.2. Exclusion Criteria

Patients with obstruction at presentation due to colon cancer were excluded. Patients who had used antibiotics two weeks prior to colonoscopy were excluded.

### 2.3. Data Collection

Demographic and clinical data were collected from all participants, including age, sex, and body mass index (BMI). Detailed medical histories, including diet, comorbidities, and the chronological order of any medications taken or procedures performed, were also obtained from each participant, and the data was entered in a Microsoft Excel file.

### 2.4. Bowel Preparation and Sample Collection

After a standard bowel preparation that included polyethylene glycol colonic preparation, a full-length colonoscopy was carried out. During the procedure, distilled water was pushed through the biopsy channel of the scope which was collected back by aspiration. The mucosal jet wash from unified segments of the colon (cecum, transverse, left, and rectal colon segments) was collected from all study participants. The healthy mucosa of each study participant was also sampled. Sites of macroscopic mucosal abnormality in any of the four selected segments were also included. Finally, 50 mL of the washes, along with the remaining colonic fluids, was aspirated through the working suction and biopsy channel. All segment samples obtained from each colonoscopy were collected in four 15 mL test tubes and immediately stored at −80°C for further processing.

### 2.5. DNA Extraction for Total Metagenomics Sequencing

All segment samples from each participant were pooled and considered representative of the whole colon to minimize technical errors and variations. DNA samples were centrifuged at 5000 ×g for 15 minutes, and the supernatants were discarded. The pellet was resuspended in 10 mL lysis buffer (0.5 M Tris-HCl; 20 mM EDTA; 10 mM NaCl; 0.1% SDS; pH 9.0), and the mixtures were homogenized by centrifuging and shaking for 5–10 minutes.

Samples were then diluted (1 : 2) with a 10 mL lysis buffer and homogenized for another 5 minutes. Genomic DNA (gDNA) was precipitated by adding 5 mL 7.5 M ammonium acetate and 25 mL ice-cold ethanol (95–100%). The samples were subsequently incubated at −20°C for 20–30 minutes, and gDNA was collected following centrifugation at 4500 ×g for 15 minutes at room temperature. DNA pellets were resuspended in 600 *μ*L TE buffer (Tris-EDTA, pH 8.0) and incubated at 65°C for 15 minutes. An equal volume of phenol-chloroform : isoamyl alcohol solution was briefly mixed with the DNA, and the mixtures were centrifuged at 12,000 ×g for 5 minutes at room temperature. The supernatant aqueous phase was then transferred to a new tube, while the interface and the organic phase were discarded. This step was repeated until no protein was visible at the interface. The final supernatant aqueous phase was then transferred to a new tube; twice the amount of ethanol was added to the aqueous phase, and the solution was stored overnight. The following day, the DNA was ethanol precipitated as described above. The resulting DNA pellet was resuspended in 50 *μ*L TE buffer. The quality and concentration of the extracted gDNA were verified using 1% agarose gel electrophoresis and a Qubit fluorometer 3.0 (Life Technologies, Carlsbad, CA).

### 2.6. Metagenomics DNA Library Construction and Sequencing

Paired-end (PE) metagenomics DNA library construction was performed, based on the manufacturer's instructions (Sequencing Kits and Reagents, Illumina, San Diego, CA). High-quality reads were separated from low-quality reads with “N” bases, adapter contamination, or human DNA contamination from the Illumina raw data using the BWA-SW algorithm (Li H, Durbin R. 2010, Bioinformatics). On average, the proportion of high-quality reads in all samples was approximately 98.1%, and the insert sizes of our PE clones ranged from 313 bp to 381 bp. Sequencing and data processing were performed at the Beijing Genomics Institute, where Illumina GAIIx and HiSeq 2000 platforms were utilized to sequence the samples.

### 2.7. Gene Catalog Construction

The first 72 randomly chosen samples were combined to establish the nonredundant gene set. Predicted open reading frames (ORFs) of the 72 samples were aligned to each other, and gene pairs with higher than 95% identity were grouped. Groups with similar genes were merged, and the longest ORF in each group was used to represent that group. We therefore organized the nonredundant gene set from all predicted genes by excluding redundant ORFs. For 159 high-quality reads in stages I, II, and III, we performed de novo assembly and gene predictions using SOAPdenovo v1.06 (Luo et al.: “SOAPdenovo2: An Empirically Improved Memory-Efficient Short-Read De Novo Assembler”. GigaScience 2012 1 : 18) and GeneMark v2.7 (Ter-Hovhannisyan, Vardges, et al. “Gene Prediction in Novel Fungal Genomes Using an Ab Initio Algorithm with Unsupervised Training”; Genome Research 18.12 (2008): 1979–1990.), respectively. All predicted genes were aligned pair-wise using BLAT, and genes that could be aligned (>90% of gene length) to another gene with more than 95% identity (no gaps allowed) were removed as redundancies, resulting in a nonredundant gene catalog. This catalog of colonic samples was further combined with the previously constructed Meta HIT gene catalog by eliminating redundancies in the same manner.

### 2.8. Bioinformatics Analysis Pipeline

**Figure 1:**
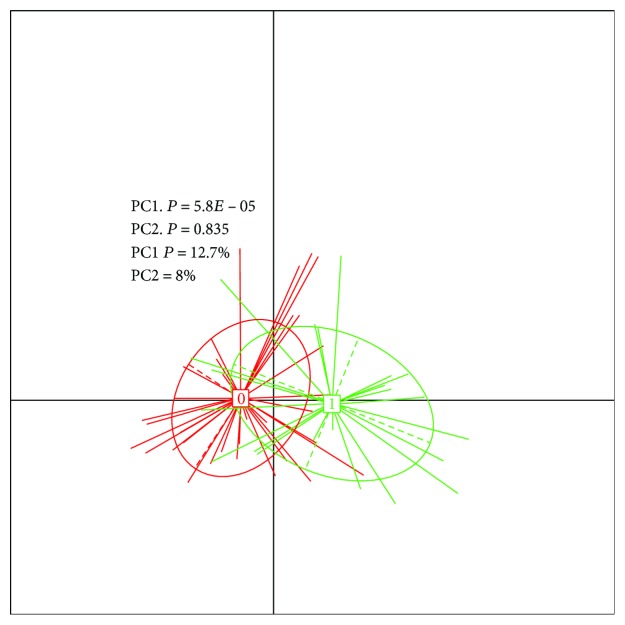
Principal coordinate analysis (PCoA) between cases and controls.

We subjected multiple samples from the mucosal microbiota metagenome to comparative phylogenetic analyses to understand the ecology of cultivation-independent gut microbiota and the phylogenetic differences between samples (Figure 1). We aligned all high-quality reads to known bacterial, fungal, protozoal, or human gut gene databases from NCBI, RDP, or MetaHIT. For each sample, we compared paired alignment and single alignments to the databases.

### 2.9. Phylogenetic Classification of ORFs

Taxonomic assignments were performed with the BLASTp alignment tool against the NR90 database. Alignment hits with *E* values greater than 1E-5 were removed, and significant matches with *E* values in the same order of the top hit were used for determining taxonomic groups. We assessed the taxonomic association of each gene by a lowest common ancestor- (LCA-) based algorithm implemented in MEGAN43.

### 2.10. Statistical Analysis

The module extracted from the ipath (reference) test was assessed by means of the Wilcoxon test between the control and the cancer groups. The Chao1 Richness Index, Shannon Index, and Simpson Diversity Index were used to describe the *α* diversity features of our bacterial community. A *P* value of <0.05 was considered significant.

Beta diversity was used to assess diversity between cases and controls. Principal coordinate analysis (PCoA) based on the unweighted UniFrac distance metrics was used to demonstrate that there was a difference in the mucosal bacterial communities between the cases and controls, which was confirmed by permutational multivariate analysis of variance (PERMANOVA) PCoA in the CRC patients.

## 3. Results

Thirty-two patients were enrolled for the CRC metagenomics study to begin with, but of these, three were excluded because of low DNA quantity in their samples. Finally, the data on 29 confirmed patients with CRC were analyzed along with a group of age- and gender-matched controls. In this study, there were 14 females and 15 males aged from 38 to 77 years.

The majority 11 (37%) of our patients were overweight (BMI = 25–30). Five (24%) patients were assigned to obesity class I (BMI = 35–40). Obesity class III BMI > 40 was observed in a 54-year-old male subject. Only 4 (13%) patients had normal BMI (BMI = 18–25). One patient in our study was observed to be mildly thin (BMI = 17-18.5), a 66-year-old female who had a BMI of just 15.66 as shown in Table 1.

Rectal bleeding was the commonest (18/29, 62%) presentation characteristic in the CRC group, and 11/29 or 37% of patients presented with symptomatic anemia. On colonoscopic examination, the CRC location was rectal in 14 (48%), and cecal growth was observed in 8 (27%). There was also hepatic flexure growth in 1 (3%) and descending colonic growth in 2 (6%), and 4 (13%) patients had transverse colon growth (Table 1). None of our patients had a familial colon cancer syndrome or any family history of colon cancer.

The histology of CRC was that of adenocarcinoma which was confirmed by two histopathologists experienced in GI histology in all patients.

The metagenomics analysis was carried out in cases and control as shown in Table 2, and in total, 3.58 G reads were sequenced, with about 321.91 G data being used in the analysis. The useful reads in each sample were aligned to 4.3 M gene set 1 by soap 2. On average, 70.33% reads can be mapped to the gene set; the max ration can reach 81.36%.

**Figure 2:**
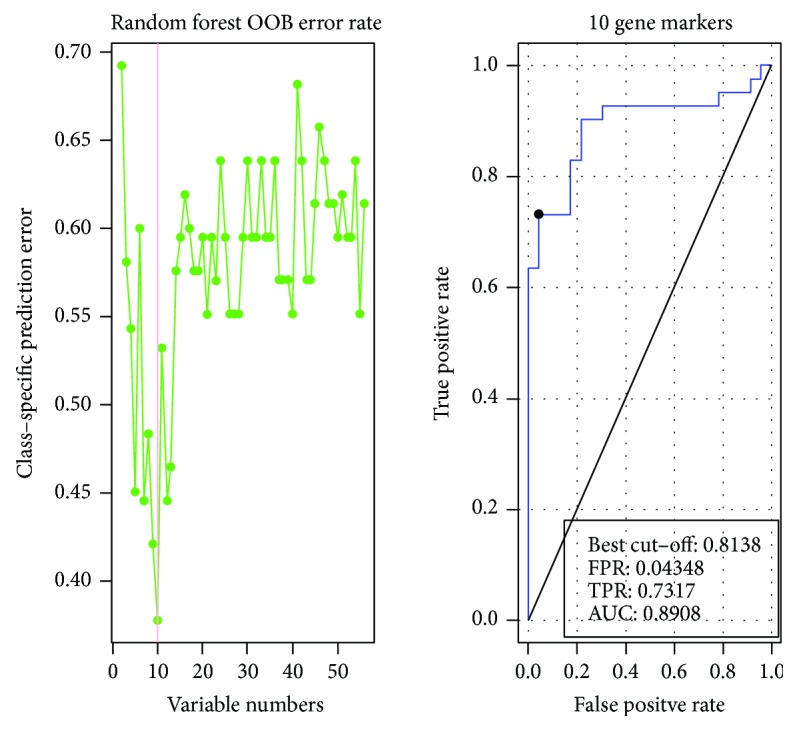
Classification based on mOTU marker in CRC cases and controls.

**Figure 3:**
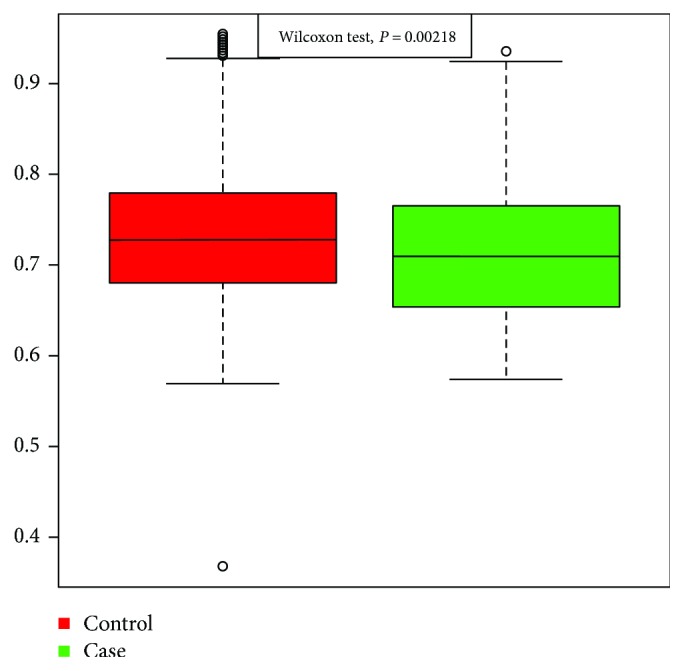
Alpha-Shannon Index in CRC cases and controls.

The Chao1 Richness Index, Shannon Index, and Simpson Diversity Index used to describe the *α* diversity features of our bacterial community are shown in Figures 2 and 3. The module extracted from the ipath (reference) test by the Wilcoxon test between the CRC and the control groups was 0.23.

All the results were mapped to 9.9 M gene sets 9 and 10. The difference between the groups in rarefaction was quite significant, as shown in Figure 1. However, the Shannon alpha diversity showed no significant difference between the diseased group and the control group, as shown in Figure 3.

Beta diversity showed significant diversity between cases and controls. Principal coordinate analysis (PCoA) based on the unweighted UniFrac distance metrics demonstrated that there was a separation in the mucosal bacterial communities between the cases and controls, which was confirmed by permutational multivariate analysis of variance (PERMANOVA) PCoA in colorectal cancer, as shown in Figure 1.

**Figure 4:**
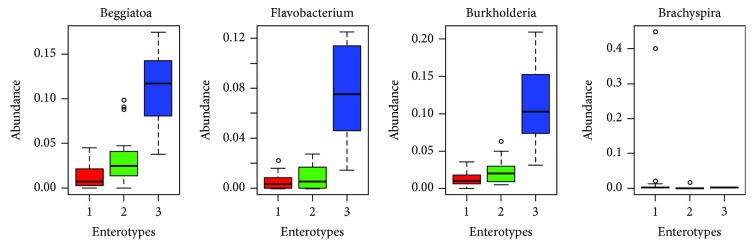
Principal coordinate analysis (PCA) in colorectal cancer patients.

It was further observed that the CRC group had statistically significantly higher 11 genera compared to those in the controls, as shown in Table 3. These genera were *Atopobium*, *Beggiatoa*, *Burkholderia*, *Collinsella*, *Comamonas*, *Finegoldia*, *Fusobacterium*, *Gemella*, *Listeria*, *Methanobrevibacter*, *Parvimonas*, *Peptoniphilus*, *Peptostreptococcus*, *Porphyromonas*, *Selenomonas*, *Shuttleworthia*, *Solobacterium*, *Thermoanaerobacter*, *Verrucomicrobiales*, and *Yersinia.* The enrichment of bacteria in colorectal cases is shown in Figure 4.

**Figure 5:**
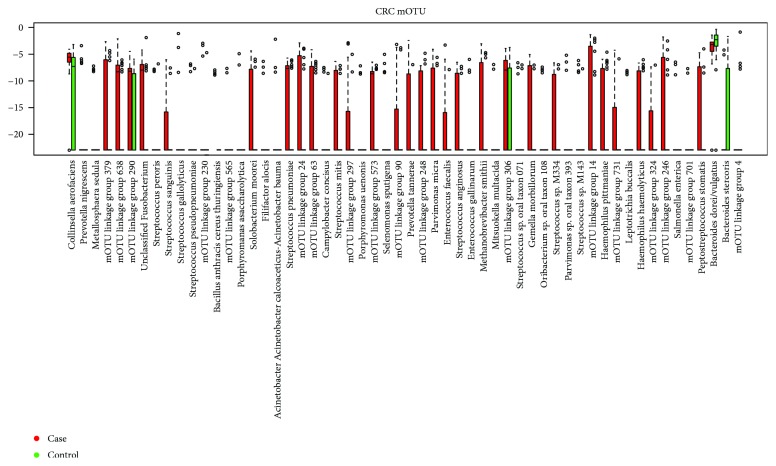
Control versus colorectal cancer case marker in mOTU.

Further, the subanalysis of Enterobacteriaceae revealed that *enterotype* 1 was observed in 15 patients with CRC while *enterotype* 2 was found to be present in 2 cases. There were six patients with *enterotype* 3 positive among the CRC group. *Enterotype* 1 was observed to be more frequently present in the control subjects than in the CRC group (Figure 5).

## 4. Discussion

Carcinogenesis in colorectal cancer (CRC) represents a heterogeneous process with a differing set of somatic molecular alterations and can be influenced by a diet and environmental and microbial exposures. Recent evidence has shown a significant link between CRC and microbiota thus affirming the old ties of bacteria and colorectal carcinogenesis observed in the past. Various studies have indicated that the presence in the gut of *Bacteroides vulgatus*, *Bacteroides stercoris*, and Clostridia species have all been directly linked to a high risk of CRC [3]. It is hypothesized that some intestinal bacteria potentiate intestinal carcinogenesis by producing genotoxins, altering the immune response and intestinal microenvironment, and activating oncogenic signaling pathways [11].

In this study, we intentionally choose metagenomic studies on colonic washes rather than on fecal samples as the evidence in support of mucosal microbiota mapping is profound. The mucosal microbiota maintains a closer interaction with the intestinal epithelium than that of the microbiota found in feces, and there is significant intersubject variability as well as differences between stool and mucosa community composition as has been demonstrated by Eckburg et al. [12].

The standard bowel prepration used in subjects in this study may be presumed to have altered the diversity of mucosa associated microbiota. Nevertheless, Harrell et al. observed that the taxonomic classification did not reveal significant changes at the phylum level, but only at the genus level. The authors of this study concluded that the degree of change underscores the importance of the need to consider the potentially influential effects of bowel preparation in experimental studies [13].

Our study showed that the CRC cases had significant enrichment of eleven genera compared to those in the control group, as shown in Table 3. The metagenomic sequencing showed that specific species, such as *Fusobacterium nucleatum*, *Peptostreptococcus stomatis*, and *Parvimonas micra*, were present in significantly greater quantities in the CRC patients than in the controls. *Fusobacterium nucleatum* has been identified to have a tumor-based immune evasion mechanism that is bacteria-dependent in the pathogenesis of CRC. Gur et al. have demonstrated that *Fusobacterium nucleatum*-bound tumors are protected from NK-mediated killing and immune cell attack due to an interaction between the fusobacterial protein Fap2 and the immune cell inhibitory receptor TIGIT on tumor-infiltrating lymphocytes including natural killer cells [14]. The multiplication of this genus in our population may possibly be a pointer to one of the target microbes for study in the future.

Another organism found to be quite significantly present in our study was *P. anaerobius*. It interacts with a toll-like receptor 2 (TLR2) and TLR4 on colon cells to increase the levels of reactive oxidative species, which promotes cholesterol synthesis and cell proliferation. Ni et al. have shown that the levels of *P. anaerobius* were found to be higher in human colon tumor tissues and adenomas when compared with nontumorous tissues. The authors of this study postulated that this bacterium increases colon dysplasia in a mouse model of CRC [15].

In animal studies, germ-free mice fed with stool from individuals with CRC developed significantly higher proportions of high-grade dysplasia (*P* < 0.05) and macroscopic polyps (*P* < 0.01) than mice fed stools from controls [16]. This suggests that the fecal microbiota from patients with CRC can promote tumorigenesis in germ-free mice, connoting the CRC and microbiota relationship.

*Fusobacterium*, *Selenomonas*, and *Peptostreptococcus* were other genera which were present in significant quantities in our CRC patients. These butyrate-producing bacteria have been identified in colorectal cases by Hibberd et al. as well [17]. The presence of Firmicutes, Proteobacteria, and Bacteroidetes was again significantly higher in CRC patients as compared to controls, as shown in Table 3. In a study by Xu and Jiang [18], microbiota in the normal, cancer, and adenoma groups were observed. The authors found that bacteria with potential tumorigenesis, like *Bacteroides fragilis* and *Fusobacterium*, were more common in the carcinoma group, while some short-chain fatty acids (SCFA) producing microbes were more numerous in the healthy group. The commensal *Escherichia* were more abundant in the adenoma patients in their study. Authors describing the same research proposed that some bacteria, such as *Butyricicoccus*, *E. coli*, and *Fusobacterium* could possibly be used as potential biomarkers for the normal, adenoma, and cancer groups, respectively.

The majority of our study participants were obese. Obesity has been linked to colon cancer and also to diabetes. The role of microbiota in both of these conditions has been described [19]. It is possible that a sinister relationship between the two exists, and bearing in mind the global epidemics of obesity and diabetes, it may be prudent to mention that to mitigate the consequences of colon cancer, both these modifiable factors should be addressed quite aggressively.

Genetic differences in intestinal microbiota in CRC patients were demonstrated by Goyal et al. [20], and the results of the current study identified changes in 11 genera in this sample of Saudi Arabian CRC patients. The limitation of our study is that the sample size was small but it nevertheless provides insights into the possible involvement of these gut microbes in CRC patients in this part of the globe.

The other side of the coin is whether knowledge of the potential role of microbiota in triggering CRC could suggest some protective interventions in colorectal carcinogenesis in the future. To address this issue, Hibberd et al. [17] observed how probiotic LGG exerted its beneficial effects and decreased the rate of CRC development. This probiotic intervention targeting microbiota could be used in humans in conjugation with other dietary supplements or drugs as part of prevention strategies for early-stage colon cancer, after further clinical validations.

To conclude, it is well known that CRC is one of the most treatable cancers, with a 5-year survival rate of approximately 64% [21]. These insights into the relationship between the microbiome, host genotype, and inflammation could suggest strategies for early diagnosis, preventive measures, and curative therapies for CRC. Further, it is anticipated that the study of microbiome dysbiosis may facilitate clinical application in CRC patient care. Hence, this study may be seen as a potential reference in this field when diagnostic tests for the early diagnosis of CRC, based on the analysis of gut microbiota, are finally discovered.

## Figures and Tables

**Table 1:** Demographic data of study participants.

Demographic data	Age range	38–77 years
	Gender ratio M : F	15 : 14
	BMI > 30	11 (37%)

Symptoms at presentation	Rectal bleeding	18/29 (62%)
	Anemia	11/29 (37%)

Location of tumor	Rectal growth	14 (48%)
	Descending colon growth	2 (6%)
	Transverse colon growth	4 (13%)
	Hepatic flexure growth	1(3%)
	Cecal growth	8 (27%)

**Table 2:** Metagenomic analysis in cases and controls.

Genus, phylum, class, order, family	*P* value	Mean rank sum		E
		Controls	Cases	
Abiotrophia, Firmicutes, Bacilli, Lactobacillales, Aerococcaceae	0.0066	24	37	0
Acidaminococcus, Firmicutes, Negativicutes, Selenomonadales, Acidaminococcaceae	0.0082	24	37	0
Akkermansia, Verrucomicrobia, Verrucomicrobiae, Verrucomicrobiales, Verrucomicrobiaceae	0.0005	22	38	0
Alcaligenes, Proteobacteria, Betaproteobacteria, Burkholderiales, Alcaligenaceae	0.0066	36	31	1
Anaerostipes, “Firmicutes,” Clostridia, Clostridiales, Lachnospiraceae	0.0005	22	38	0
Azoarcus, Proteobacteria, Betaproteobacteria, Rhodocyclales, Rhodocyclaceae	0.0086	39	29	1
Bacteroides, Bacteroidetes, Bacteroidia, Bacteroidales, Bacteroidaceae	0.0072	24	37	0
Beggiatoa, Proteobacteria, Gammaproteobacteria, Thiotrichales, Thiotrichaceae	0.0003	44	26	1
Burkholderia, Proteobacteria, Betaproteobacteria, Burkholderiales, Burkholderiaceae	0.0028	42	27	1
Butyrivibrio, Firmicutes, Clostridia, Clostridiales, Lachnospiraceae	0.0005	22	38	0
Chlorobium, Chlorobi, Chlorobia, Chlorobiales, Chlorobiaceae	0.0027	39	29	1
Clostridium, Firmicutes, Clostridia, Clostridiales, Clostridiaceae	0.0002	21	39	0
Coprococcus, Firmicutes, Clostridia, Clostridiales, Lachnospiraceae	0.0052	24	37	0
Coriobacterium, Actinobacteria, Actinobacteria, Coriobacteriales, Coriobacteriaceae	0.0083	38	29	1
Coxiella, Proteobacteria, Gammaproteobacteria, Legionellales, Coxiellaceae	0.0066	36	31	1
Crenothrix, Bacteroidetes, Sphingobacteria, Sphingobacteriales, Crenotrichaceae	0.0013	42	27	1
Cryptobacterium, Actinobacteria, Actinobacteria, Coriobacteriales, Coriobacteriaceae	0.0027	39	29	1
Dethiobacter, Firmicutes, Clostridia, Clostridiales, Syntrophomonadaceae	0.0084	40	28	1
Enterobacter, Proteobacteria, Gammaproteobacteria, Enterobacteriales, Enterobacteriaceae	0.0004	22	39	0
Eubacterium, Firmicutes, Clostridia, Clostridiale, Eubacteriaceae	0.0026	23	38	0
Haemophilus, Proteobacteria, Gammaproteobacteria, Pasteurellales, Pasteurellaceae	0.0060	24	37	0
Haladaptatus, Archaea, Euryarchaeota, Halobacteria, Halobacteriales, Haladaptatus	0.0007	38	30	1
Holdemania, Firmicutes, Erysipelotrichi, Erysipelotrichales, Erysipelotrichidae	0.0004	22	39	0
Klebsiella, Proteobacteria, Gammaproteobacteria, Enterobacteriales, Enterobacteriaceae	0.0066	24	37	0
Listeria, Firmicutes, Bacilli, Bacillales, Listeriaceae	0.0008	41	28	1
Megasphaera, Firmicutes, Negativicutes, Selenomonadales, Veillonellaceae	0.0021	23	38	0
Mycoplasma, Tenericutes, Mollicutes, Mycoplasmatales, Mycoplasmataceae	0.0025	40	28	1
Paracoccus, Proteobacteria, Alphaproteobacteria, Rhodobacterales, Rhodobacteraceae	0.0075	41	28	1
Polaribacter, Bacteroidetes, Flavobacteria, Flavobacteriales, Flavobacteriaceae	0.0002	43	27	1
Roseburia, Firmicutes, Clostridia, Clostridiales, Lachnospiraceae	0.0057	24	37	0
Serratia, Proteobacteria, Gammaproteobacteria, Enterobacteriales, Enterobacteriaceae	0.0002	41	28	1
Sphaerochaeta, Spirochaetes, Spirochaetia, Brachyspirales, Sarpulinacea	0.0004	43	26	1
Sulfurovum, Proteobacteria, Epsilonproteobacteria	0.0027	42	27	1
Ureaplasma, Tenericutes, Mollicutes, Mycoplasmatales, Mycoplasmataceae	0.0034	39	29	1
Unclassified	0.0031	23	38	0

**Table 3:** Significant enrichment in colorectal cases.

Genus, phylum, class, order, family	*P* value	Enrichment
Atopobium, Actinobacteria, Coriobacteriia, Coriobacteriales, Coriobacteriaceae	0.00047	CRC
Beggiatoa, Proteobacteria, Gammaproteobacteria, Thiotrichales, Thiotrichaceae	0.0002	CRC
Burkholderia, Proteobacteria, Betaproteobacteria, Burkholderiales, Burkholderiaceae	0.00011	CRC
Collinsella, Actinobacteria, Actinobacteria, Coriobacteriales, Coriobacteriaceae	0.00269	CRC
Comamonas, Proteobacteria, Betaproteobacteria, Burkholderiales, Comamonadaceae	0.00665	CRC
Finegoldia, Firmicutes, Clostridia, Clostridiales, Peptoniphilaceae	0.00726	CRC
Fusobacterium, Firmicutes, Clostridia, Clostridiales, Peptoniphilaceae	0.00751	CRC
Gemella, Firmicutes, Bacilli, Bacillales	0.00743	CRC
Listeria, Firmicutes, Bacilli, Bacillales, Listeriaceae	0.00657	CRC
Methanobrevibacter, Euryarchaeota, Methanobacteria, Methanobacteriales, Methanobacteriaceae	7.63*E* − 05	CRC
Peptostreptococcus, Firmicutes, Clostridia, Clostridiales, Peptostreptococcaceae	0.00014	CRC
Peptoniphilus, Firmicutes, Clostridia, Clostridiales	0.00023	CRC
Peptostreptococcus, Firmicutes, Clostridia, Clostridiales, Peptostreptococcaceae	0.00142	CRC
Porphyromonas, Bacteroidetes, Bacteroidetes, Bacteroidales, Porphyromonadaceae	0.0066	CRC
Selenomonas, Firmicutes, Negativicutes, Selenomonadales, Veillonellaceae	0.00343	CRC
Solobacterium, Firmicutes, Erysipelotrichi, Erysipelotrichales, Erysipelotrichidae	0.00904	CRC
Thermoanaerobacter, Firmicutes, Clostridia, Thermoanaerobacterales, Thermoanaerobacteraceae	0.00043	CRC
Verrucomicrobiales, Verrucomicrobia, Verrucomicrobiae, Verrucomicrobiales, Verrucomicrobiacea	1.68*E* − 06	CRC
Yersinia, Proteobacteria, Gammaproteobacteria, Enterobacteriales, Yersiniacea	0.0095	CRC
